# Small RNA-seq analysis in response to methyl jasmonate and abscisic acid treatment in *Persicaria minor*

**DOI:** 10.1016/j.gdata.2017.05.011

**Published:** 2017-05-12

**Authors:** Nazaruddin Nazaruddin, Abdul Fatah A. Samad, Muhammad Sajad, Jaeyres Jani, Zamri Zainal, Ismanizan Ismail

**Affiliations:** a School of Biosciences and Biotechnology, Faculty of Science and Technology, Universiti Kebangsaan Malaysia, 43600 Bangi, Selangor, Malaysia; b Institute of Systems Biology (INBIOSIS), Universiti Kebangsaan Malaysia, 43600 Bangi, Selangor, Malaysia; c Department of Chemistry, Faculty of Mathematics and Natural Sciences, University of Syiah Kuala, Darussalam, Banda Aceh 23111, Indonesia; d Department of Plant Breeding and Genetics, University College of Agriculture &Environmental Sciences, The Islamia University of Bahawalpur, Punjab, Pakistan; e BioEasy Sdn. Bhd. and ScienceVision Sdn. Bhd., Setia Alam, Seksyen U13, 40170 Shah Alam, Selangor, Malaysia

**Keywords:** *Persicaria minor*, Small RNA sequencing, miRNA, Methyl jasmonate and abscisic acid

## Abstract

*Persicaria minor* (Kesum) is an important medicinal plant with high level of secondary metabolite contents, especially, terpenoids and flavonoids. Previous studies have revealed that application of exogenous phytohormone could increase secondary metabolite contents of the plant. MicroRNAs (miRNAs) are small RNAs that play important regulatory roles in various biological processes. In order to explore the possible role of miRNA in the regulation of these phytohormones signaling pathway and uncovering their potential correlation, we, for the first time, have generated the smallRNA library of Kesum plant. The library was developed in response to methyl jasmonate (MJ) and abscisic acid (ABA) treatment by using next-generation sequencing technology. Raw reads have been deposited to SRA database with the accession numbers, SRX2655642 and SRX2655643 (MJ-treated), SRXSRX2655644 and SRX2655645 (ABA-treated) and SRX2655646and SRX2655647 (Control).

**Table:** 

Specifications	
Organism/cell line/tissue	*Persicaria minor* (leaf)
Sex	Not applicable
Sequencer or array type	Illumina HiSeqTM 2500 in Rapid Run mode
Data format	FASTQ
Experimental factors	MJ and ABA treatments
Experimental features	Controlled growth chamber set for 25 ± 2 °C with photoperiod of 16 h of light and 8 h of darkness
Consent	Not applicable
Sample source location	Selangor, Malaysia; GPS coordinates: (3° 16′14.63″ N, 101° 41′ 11.32″ E)

## 1 Direct link to deposited data

https://www.ncbi.nlm.nih.gov/sra/SRX2655642; (MJ-treated).

https://www.ncbi.nlm.nih.gov/sra/SRX2655643; (MJ-treated).

https://www.ncbi.nlm.nih.gov/sra/SRX2655644; (ABA-treated).

https://www.ncbi.nlm.nih.gov/sra/SRX2655645; (ABA-treated).

https://www.ncbi.nlm.nih.gov/sra/SRX2655646; (Control).

https://www.ncbi.nlm.nih.gov/sra/SRX2655647; (Control).

## 2 Experimental design, materials and methods

### 2.1 Plant materials

*Persicaria minor* plants were grown on an MS solid medium in a growth chamber at 25 ± 2 °C with a photoperiod of 16 h of light and 8 h of darkness. Healthy, 45 day plants were selected for MJ and ABA elicitation, and leaves were sprayed with 100 μM of MJ and 100 μM of ABA, while the control plants were sprayed with distilled water. For MJ-treatment, leaves sample were harvested after 2 days (maximum gene expression was previosly reported after 2 days) [1], whereas; samples for ABA-treatment were harvested after 3 days. The harvested samples were blotted dry, frozen in liquid nitrogen and stored at − 80 °C for further total RNA extraction.

### 2.2 Total RNA extraction, quality control, library preparation and small RNA-seq

Total RNA from leaf samples were extracted using Plant RNA purification reagent (Invitrogen, USA) based on manufacturer's protocol. Quantitation of extracted total RNA was carried out using Nanodrop 1000 (Thermo Fisher Scientific Inc., USA) whereas, the integrity of was determined by Agilent 2100 Bioanalyzer (Agilent Technology, USA), respectively. Total RNAs with a RIN value > 7 were selected for library construction. NEBNext® Multiplex Small RNA Library Prep Set for Illumina® (Set 1) Kit was used for library construction using the manufacturer's protocol.

Two biological replicates were prepared for each treatment. The small-RNA samples were sequenced using the Illumina HiSeq 2500 platform. Reads with 50 bp (single-end read) were generated for individual samples.

### 2.3 Raw reads processing, annotation and classification

CLC Genomic Workbench version 8 (https://www.qiagenbioinformatics.com/) was used to analyze the data. The analysis was carried out by removing adaptor and low quality sequences. The remaining sequences were filtered and the sequences between 18 bp to 30 bp were obtained (Table 1). The filtered sequences were considered for annotation. Firstly, the sequences were mapped against 73 plant species data from miRBase version 21 [2]. The sequences with no hit in the miRBase were used for further annotation against Rfam database (Table 2) for non-coding RNAs (tRNA, snoRNA, SnRNA, and rRNA) [3], [4]. The remaining sequences having no hit with the above databases were categorized as unannotated.

**Table 1:** Raw reads pre-analysis.

SRA ID	Total number of reads	Number of reads after trimming	Discarded reads
SRX2655642 (MJ-treated)	33,496,292	19,094,706	14,401,586
SRX2655643 (MJ-treated)	47,378,860	23,823,127	23,555,733
SRX2655644 (ABA-treated)	3,940,941	2,459,779	1,481,162
SRX2655645 (ABA-treated)	30,566,191	20,683,762	9,882,429
SRX2655646 (Control)	11,223,189	6,609,315	4,613,874
SRX2655647 (Control)	26,046,620	15,337,046	10,709,574

**Table 2:** Raw reads annotation analysis.

SRA ID	Total number of sequence tags	Annotation by miRBase	Annotation by Rfam	Unannotated
SRX2655642 (MJ-treated)	1,923,235	2899	110,834	1,809,502
SRX2655643 (MJ-treated)	2,403,188	2646	1268,691	2,271,792
SRX2655644 (ABA-treated)	496,306	231	36,572	459,503
SRX2655645 (ABA-treated)	2,665,163	845	101,141	2,563,177
SRX2655646 (Control)	1,194,072	1548	74,274	1,118,250
SRX2655647 (Control)	2,511,221	700	93,975	2,416,546

### 2.4 Differential miRNA expression analysis

The expression of miRNAs in treated and control libraries was compared. To proceed with the differential expression analysis, the first step in the statistical analysis was the normalization of the data to transcripts per million (TPM) which was done using criterion: TPM = (actual miRNA count/total count of clean reads) ∗ 1,000,000. After the data normalization, the fold change (Fold change = log2 (miRNA TPM in the treatment library/miRNA TPM in the control library) and p-value was calculated using Baggerly's test [5]. Finally, differentially expressed miRNAs were filtered using False Discovery Rates (FDR) ≤ 0.05 and the absolute value log_2_ ratio ≥ 1. Table 3 showed the number of miRNAs that significantly affected by both treatment.

**Table 3:** Differential miRNAs expression analysis.

Treatments	Total miRNAs significantly affected	Number of miRNAs upregulated	Number of miRNAs downregulated
MJ treatment	301	273	28
ABA treatment	33	4	29

## Conflict of interest

All the authors have approved submission and there are no conflicts of interest.
